# Genome reduction in novel, obligately methyl-reducing *Methanosarcinales* isolated from arthropod guts (*Methanolapillus* gen. nov. and *Methanimicrococcus*)

**DOI:** 10.1093/femsec/fiae111

**Published:** 2024-08-06

**Authors:** Evgenii Protasov, Hanna Reeh, Pengfei Liu, Anja Poehlein, Katja Platt, Thomas Heimerl, Vincent Hervé, Rolf Daniel, Andreas Brune

**Affiliations:** Research Group Insect Gut Microbiology and Symbiosis, Max Planck Institute for Terrestrial Microbiology, 35043 Marburg, Germany; Microcosm Earth Center, Max Planck Institute for Terrestrial Microbiology and Philipps-Universität Marburg, 35043 Marburg, Germany; Research Group Insect Gut Microbiology and Symbiosis, Max Planck Institute for Terrestrial Microbiology, 35043 Marburg, Germany; Research Group Insect Gut Microbiology and Symbiosis, Max Planck Institute for Terrestrial Microbiology, 35043 Marburg, Germany; Center for Pan-third Pole Environment, Lanzhou University, 730000 Lanzhou, China; Genomic and Applied Microbiology and Göttingen Genomics Laboratory, Institute of Microbiology and Genetics, Georg-August-University Göttingen, 37077 Göttingen, Germany; Research Group Insect Gut Microbiology and Symbiosis, Max Planck Institute for Terrestrial Microbiology, 35043 Marburg, Germany; Center for Synthetic Microbiology (SYNMIKRO), 35043 Marburg, Germany; Research Group Insect Gut Microbiology and Symbiosis, Max Planck Institute for Terrestrial Microbiology, 35043 Marburg, Germany; Université Paris-Saclay, INRAE, AgroParisTech , UMR SayFood, 91120 Palaiseau, France; Genomic and Applied Microbiology and Göttingen Genomics Laboratory, Institute of Microbiology and Genetics, Georg-August-University Göttingen, 37077 Göttingen, Germany; Research Group Insect Gut Microbiology and Symbiosis, Max Planck Institute for Terrestrial Microbiology, 35043 Marburg, Germany

**Keywords:** archaea, arthropods, cockroaches, gut microbiota, methanogens, millipedes, termites

## Abstract

Recent metagenomic studies have identified numerous lineages of hydrogen-dependent, obligately methyl-reducing methanogens. Yet, only a few representatives have been isolated in pure culture. Here, we describe six new species with this capability in the family *Methanosarcinaceae* (order *Methanosarcinales*), which makes up a substantial fraction of the methanogenic community in arthropod guts. Phylogenomic analysis placed the isolates from cockroach hindguts into the genus *Methanimicrococcus* (*M. hacksteinii, M. hongohii*, and *M. stummii*) and the isolates from millipede hindguts into a new genus, *Methanolapillus* (*M. africanus, M. millepedarum*, and *M. ohkumae*). Members of this intestinal clade, which includes also uncultured representatives from termites and vertebrates, have substantially smaller genomes (1.6–2.2 Mbp) than other *Methanosarcinales*. Genome reduction was accompanied by the loss of the upper part of the Wood–Ljungdahl pathway, several energy-converting membrane complexes (Fpo, Ech, and Rnf), and various biosynthetic pathways. However, genes involved in the protection against reactive oxygen species (catalase and superoxide reductase) were conserved in all genomes, including cytochrome *bd* (CydAB), a high-affinity terminal oxidase that may confer the capacity for microaerobic respiration. Since host-associated *Methanosarcinales* are nested within omnivorous lineages, we conclude that the specialization on methyl groups is an adaptation to the intestinal environment.

## Teaser

New methanogens from arthropod guts demonstrate significant genome reduction but also presence of high-affinity terminal oxidases.

## Introduction

Evidence for the presence of obligately methyl-reducing methanogens in many so-far uncultured archaeal lineages has sparked debate about the implications for the evolutionary origin of methanogenesis (Borrel et al. 2019, Wang et al. 2021, Mei et al. 2023). However, only six species with this capability have been isolated, which limits biochemical and physiological experiments to elucidate their metabolic features. The isolates fall into four distantly related orders and belong to the genera *Methanosphaera* (order *Methanobacteriales*), *Methanomassiliicoccus* and *Methanomethylophilus* (order *Methanomassiliicoccales*), *Methanonatronarchaeum* (order *Methanonatronarchaeales*), and *Methanimicrococcus* (order *Methanosarcinales*).

Members of the order *Methanosarcinales* are morphologically, biochemically, and genomically distinct from other methanogens and occur in a wide range of environments, such as marine sediments, wetlands, soils, and the intestinal tracts of animals. They show the widest substrate range of all methanogens, including not only the classical substrates CO_2_, methanol, and acetate but also methylated sulfur compounds and methoxylated aromatic compounds (Kurth et al. 2020). In contrast to other methanogens, *Methanosarcinales* possess cytochromes and membrane-associated electron transport chains, resulting in the highest growth yields among methanogens (Thauer et al. 2008, Mand and Metcalf 2019).

The only cultured representative of *Methanosarcinales* from animal guts is *Methanimicrococcus blatticola*, isolated from the hindgut of the cockroach *Periplaneta americana* (Sprenger et al. 2000). It differs from other members of *Methanosarcinales* in its restriction to methanol or methylamines as methanogenic substrates, an obligate dependency on external H_2_ resulting from the inability to oxidize methyl groups to CO_2_, and a requirement for several growth supplements including acetate and coenzyme M (CoM). Comparative genome analysis revealed that *M. blatticola* has a highly reduced genome and lacks essential elements of the methanogenic pathways present in other *Methanosarcinales*, including the Wood–Ljungdahl pathway and many membrane-associated complexes (Thomas et al. 2021), which explains the limited substrate spectrum and growth requirements documented in earlier studies (Sprenger et al. 2000, 2005).

*Methanimicrococcus*-related sequences have also been detected in 16S rRNA-based studies of other cockroaches, termites (the closest relatives of cockroaches), and scarab beetle larvae (Ohkuma et al. 1999, Friedrich et al. 2001, Egert et al. 2003), and more recently also in millipedes (Protasov et al. 2023). Together with sequences from vertebrate guts, they form a monophyletic clade that consists exclusively of representatives from the intestinal tract of animals (Thomas et al. 2022, Protasov et al. 2023).

Here, we report the isolation and physiological characterization of six new species of obligately methyl-reducing methanogens from the hindgut of cockroaches and millipedes. Based on a previous, phylogenomic analysis of archaeal diversity in arthropods, which covered both isolates and uncultured lineages, the isolates were already described under the *Code of Nomenclature of Prokaryotes Described from Sequence Data* (SeqCode) (Hedlund et al. 2022) as new species of the genera *Methanimicrococcus* and *Methanolapillus* (Protasov et al. 2023). In the present study, we provide a detailed characterization of these taxa and their formal description under the *International Code of Nomenclature of Prokaryotes* (ICNP) (Oren et al. 2023), together with a comparative analysis of all available genomes of host-associated *Methanosarcinales* that includes uncultured representatives from termites and vertebrates.

## Materials and methods

### Microbiological medium

Microorganisms were isolated and routinely cultivated in anoxic bicarbonate-buffered mineral medium AM7 supplemented with vitamins and other growth factors. The medium contained (per liter) 1 g NaCl, 0.4 g MgCl_2_·6H2O, 0.1 g CaCl_2_·2H_2_O, 0.3 g NH_4_Cl, and 0.2 g KH_2_PO_4_; 1 ml trace element solution SL 10 (1.5 g FeC1_2_·4H_2_O, 2 mg CuCl_2_·2H_2_O, 24 mg NiCl_2_·6H_2_O, 36 mg Na_2_MoO_4_·2H_2_O, 70 mg ZnCl_2_, 100 mg MnCl_2_·4 H_2_O, 190 mg CoCl2·6H_2_O, 6 mg H_3_BO_3_ per liter), 1 ml selenite–tungstate solution (0.5 g NaOH, 3 mg Na_2_SeO_3_·5H_2_O, 4 mg Na_2_WO_4_·2H_2_O per liter), 1 g yeast extract, 1 g Casamino acids, 0.1 g tryptophan (to compensate lack of tryptophan in Casamino acids), 1 g peptone, 1 g sodium acetate, and 1 g sodium formate. Resazurin (0.8 mg/l) was included as redox indicator. After autoclaving, the medium was cooled under an N_2_/CO_2_ (80:20) atmosphere, and the following supplements (final concentrations) were added from sterile stock solutions, following the procedure described by Widdel and Pfennig (1981): NaHCO_3_ (30 mM); 1 ml 7-vitamin solution (10 mg biotin, 25 mg calcium pantothenate, 50 mg thiamine, 50 mg *p*-aminobenzoic acid, 100 mg nicotinic acid, and 250 mg pyridoxamine, 50 mg vitamin B12 per liter); CoM (10 µM); menadione (vitamin K3, 2.5 µM); lipoic acid (1 µM); folic acid and riboflavin (50 µg/l each); isobutyric, 2-methylbutyric, *n*-valeric, and isovaleric acids (25 µM each); phenylacetic, phenylpropionic, 4-hydroxyphenylacetic, and 3-indolylacetic acids (5 µM each). Cysteine (1 mM) and dithiothreitol (1 mM) were added as reducing agents. Methanol (50 mM final concentration) was added from sterile stock solutions stored under N_2_ atmosphere. If necessary, the pH was adjusted to 7.0 with HCl. The medium was dispensed into 60-ml serum vials (30 ml medium) or 16-ml rubber-stoppered Hungate-type culture tubes (4.5 ml medium) and gassed with H_2_/CO_2_ (80:20).

### Enrichment and isolation

Cockroaches (*Archimandrita tessellata, Eublaberus serranus*, and *Henschoutedenia flexivitta*) and millipedes (*Atopochetus caudulanus, Archispirostreptus gigas*, and *Anadenobolus monilicornis*) were obtained from commercial breeders (Jörg Bernhardt, Glashütte, Germany, www.schaben-spinnen.de; Home of Insects, Erfurt, Germany, www.home-of-insects.com) and maintained in our laboratory as previously described (Schauer et al. 2012). Adult specimens were dissected, and the entire gut was placed in a sterile Hungate tube containing 2-mm glass beads (2 g). After addition of 10 ml AM7 medium, the tube was closed with a rubber stopper, the headspace was gassed with N_2_/CO_2_ (80:20), and the gut was homogenized by vortexing the tube for 2–5 min. The gut homogenate was inoculated into culture tubes containing 4.5 ml of AM7 medium with methanol (50 mM) under a headspace of H_2_/CO_2_ (80/20) and incubated at 30°C. Cultures that produced methane were transferred into AM7 medium with 50 mM methanol supplemented with kanamycin and ampicillin (each 100 µg/ml final concentration). Pure cultures were obtained by the isolation of individual colonies from deep-agar dilution series with 1% agar (Pfennig and Trüper 1981) using the same medium and growth conditions. The absence of bacterial contaminants was assessed by inoculating the cultures into AM7 medium with 10 mM glucose. *Methanimicrococcus blatticola* (DSM 13328) was obtained from the Deutsche Sammlung von Mikroorganismen und Zellkulturen (DSMZ, Braunschweig, Germany).

### Growth, medium requirements, and physiology

Growth was measured directly in the culture tubes by following the increase in optical density at 578 nm (OD578) using a culture tube photometer (Spectronic 20^+^, Milton Roy; path length ca. 1.3 cm). Dry weight was determined with triplicate cultures grown on methanol (50 mM) in 1-l glass vessels containing 500 ml AM7 medium under H_2_/CO_2_. After OD measurement, the cells were harvested by centrifugation (10 000 × *g*; 20 min), washed with ammonium acetate solution (20 mM), and dried at 60°C to weight constancy.

For substrate spectra, additional substrates were tested (final concentration): formate (10 mM), propanol (10 mM), ethanol (10 mM), acetate (10 mM), mono-, di-, and trimethylamine (10 mM), syringate (4-hydroxy- 3,5-dimethoxybenzoate) (10 mM), vanillate (4-hydroxy-3-methoxy-benzoate) (10 mM), and 3,4,5-trimethoxybenzoate (10 mM) were tested. Growth in the absence of H_2_ was tested under a headspace of N_2_/CO_2_ (80:20).

Other growth requirements (yeast extract, peptone, CoM, acetate, and formate) were assessed after transferring the cultures at least three times in medium lacking a particular supplement, determining growth and methane production at 7 and 14 days after inoculation. A supplement was considered as essential if no growth occurred after 14 days or as stimulatory if growth and methane production after 14 days did not exceed that in full medium after 7 days.

### Methane measurements

Aliquots of headspace gas (0.2 ml) were sampled every 7 days with a gas-tight syringe, and the methane content was analysed using a gas chromatograph (SRI 8610C) equipped with a packed column (Porapak Q, 80/100 mesh, 274 cm by 3.18 mm inside diameter) and a flame ionization detector.

### Light and electron microscopy

For phase-contrast light microscopy of unfixed cells, an Axiophot photomicroscope (Zeiss, Oberkochen, Germany) was used. For photomicrographs, cultures were applied to agar-coated slides (Pfennig and Wagener 1986). Autofluorescence of cofactor F_420_ was observed with the same microscope with a UV light source and appropriate filter sets, using *Methanobrevibacter ruminantium* DSM 1093 as positive control.

Concentrated cell suspensions (3 µl) were high-pressure frozen, freeze-substituted with an acetone solution containing 0.25% osmium tetroxide, 0.2% uranyl acetate, and 5% H_2_O), and embedded in Epon 812 substitute resin (for details, see Renicke et al. 2017). Ultrathin sections (50 nm) were cut with a microtome equipped with a diamond blade. Sections were poststained with aqueous solutions of 2% uranyl acetate and 0.5% lead citrate. The sections were examined with a JEM-2100 transmission electron microscope (JEOL, Tokyo, Japan) operated at 120 kV. Images were acquired with a F214 fast-scan CCD camera (TVPIS, Gauting, Germany).

### Genome sequencing and annotation

High molecular weight DNA from isolates was prepared with the DNAEasy Blood & Tissue Kit (Qiagen) following the manufacturer’s protocol. The procedures for genome sequencing, assembly, and annotation have been previously described (Protasov et al. 2023). Metagenome-assembled genomes were obtained from various species of termites and annotated in a previous study (Hervé et al. 2020). All genomes and 16S rRNA genes are available at NCBI GenBank (accession numbers are given in the corresponding figures and in the Taxonomy section). For the analysis of the metabolic pathways, annotation results were verified, and missing functions were identified using Blast with a threshold E-value of 1E–5 and BlastKoala (Kanehisa et al. 2016). Amino acids biosynthesis pathways were additionally checked and annotated with GapMind (Price et al. 2020).

### Phylogenetic analysis

The 16S rRNA gene sequences of the isolates and MAGs were imported into the Dictyopteran gut microbiota reference database (DictDb v. 5.1 Archaea) (Protasov et al. 2023) and aligned against the existing alignment of previously published sequences with the SINA aligner using the ARB software package (Ludwig et al. 2004, Pruesse et al. 2012). After manual curation of the alignment, a maximum-likelihood tree was calculated using IQ-TREE 2 with the substitution model GTR+I+G4 (Kalyaanamoorthy et al. 2017, Minh et al. 2020). Node support was assessed using the Shimodaira–Hasegawa approximate-likelihood ratio test (Guindon et al. 2010, Hoang et al. 2018).

The genomes from isolates and MAGs were phylogenetically classified within the taxonomic framework of the Genome Taxonomy Database (GTDB, release 207.2) using the GTDB toolkit (v. 2.1.1) (Chaumeil et al. 2020, Hervé et al. 2020). A maximum-likelihood tree was inferred from a concatenated alignment of 53 archaeal single-copy marker genes generated by GTDB toolkit using IQ-Tree with the substitution model LG+F+R4 selected by the ModelFinder tool and nonparametric bootstrap branch support (Kalyaanamoorthy et al. 2017, Rinke et al. 2021). The average nucleotide identities of the genomes were calculated with FastANI version 1.3 (Jain et al. 2018).

### Data visualization and statistical analysis

Statistical analyses were performed with R v3.5.1, and plots were generated with ggplot2 (Wickham 2016). Genome size and number of protein coding genes were tested for normality of distribution with Shapiro–Wilk normality test, Kruskal–Wallis, and *post hoc* Dunn tests with Holm *P*-value adjustment were performed using the ggbetweenstats function from the ggstatsplot package in R (Patil 2021).

The phylogenomic tree was visualized using iTOL v. 6.8 (Letunic and Bork 2021) while 16S rRNA phylogenetic tree was visualized using ARB software package (v7.0) (Ludwig et al. 2004). Phylogenetic trees and genome comparison figures were edited in Inkscape v1.3.2.

## Results

### Isolation and morphological characterization

Six strains of methyl-reducing *Methanosarcinales* were isolated from enrichment cultures on H_2_ + methanol that were inoculated with gut homogenates of the millipedes *A. caudulanus* (strain Ac7), *A. gigas* (strain Ag5), and *A. monilicornis* (strain Am2), and of the cockroaches *A. tessellata* (strain At1), *E. serranus* (strain Es2), and *H. flexivitta* (strain Hf6). The isolates were obtained from single colonies in deep-agar dilution series; the dilution series were repeated to ensure the clonality of the culture (Pfennig and Trüper 1981). All strains formed lens-shaped, cream-colored colonies that reached a diameter of up to 2 mm after 1 month of incubation (Fig. A1).

**Figure 1.**
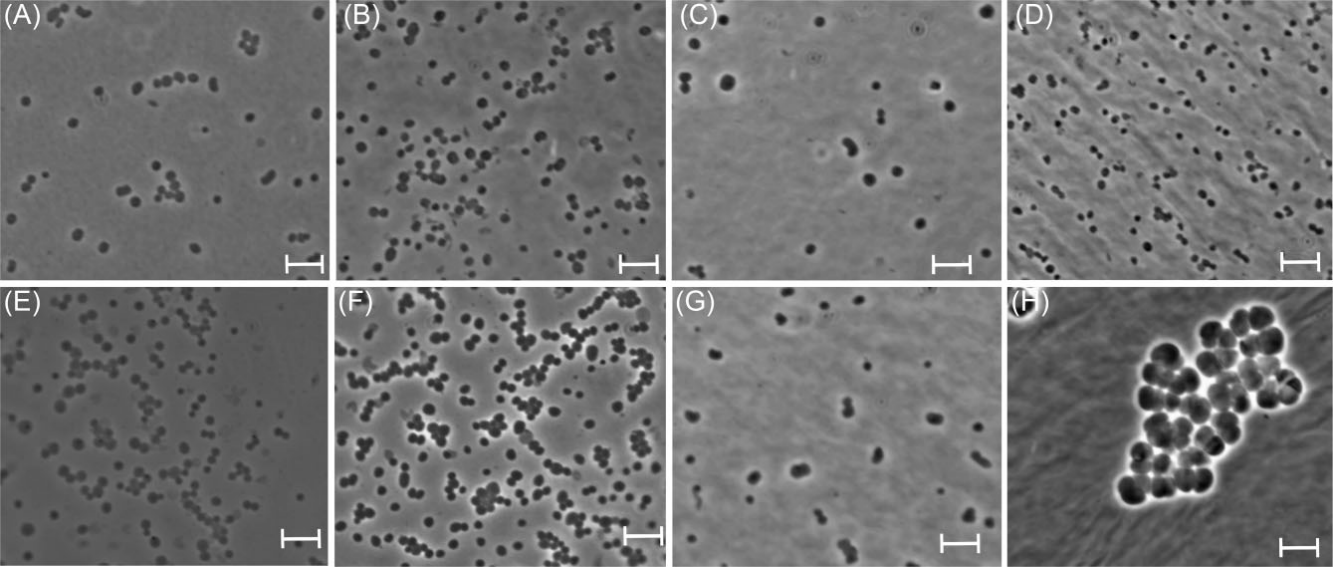
Phase-contrast photomicrographs of *Methanosarcinales* isolated from arthropod guts: *Methanimicrococcus hacksteinii* At1 (A), *Methanimicrococcus stummii* Es2 (B), *Methanimicrococcus hongohii* Hf6 (C), *Methanimicrococcus blatticola* PA (D), *Methanolapillus millipedarum* Ac7 (E), *Methanolapillus africanus* Ag5 (F), and *Methanolapillus ohkumae* Am2 (G). *Methanosarcina barkeri* MS (H) is included for comparison. Agar slides were prepared from cultures in the early exponential phase. Scale bars represent 5 µm.

**Figure 2.**
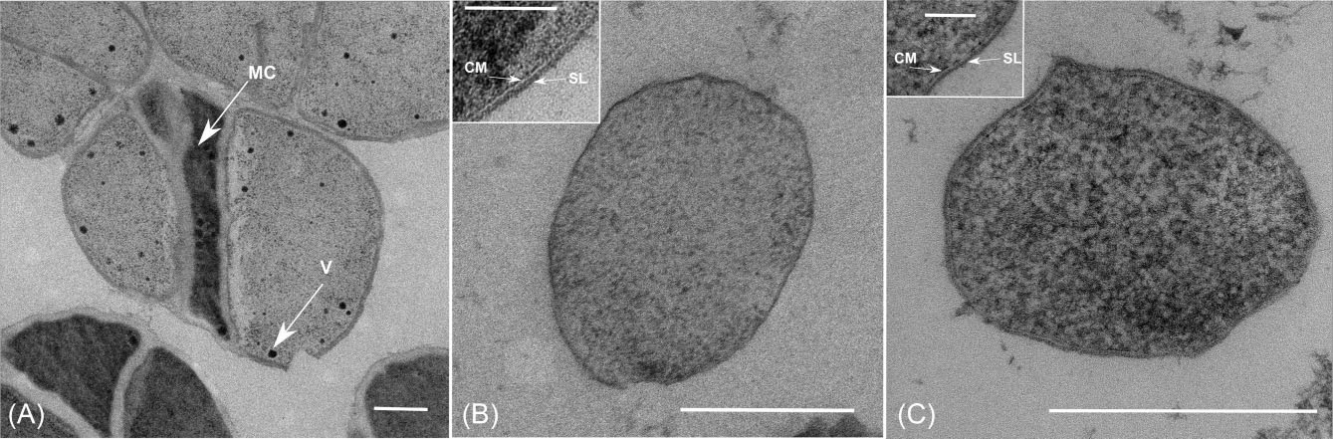
Electron micrographs of ultra-thin sections of *M. barkeri* MS (A), *M. africanus* Ag5 (B), and *M. stummii* Es2 (C). The magnified inserts show details of the cell envelope. Abbreviations: MC, methanochondroitin; CM, cytoplasmic membrane; SL, S-layer; and V, volutin granules (polyphosphate). Scale bars 0.5 µm (inserts 0.1 µm).

Phase-contrast microscopy of liquid cultures in the early exponential phase showed nonmotile, irregular cocci with a diameter of 1.0–2.0 µm that resembled the cells of *M. blatticola* (Fig. 1A–G). None of the strains formed cell clusters typical of *Methanosarcina barkeri* and some other *Methanosarcina* spp. (Fig. 1H); only small clusters of cells were observed on rare occasions. All strains exhibited the characteristic fluorescence of cofactor F_420_ in all growth phases, but it was weaker than in *Methanosarcina* spp. Ultrathin sections of strains Ag5 and Es2 revealed the presence of an S-layer surrounding the cytoplasmic membrane, but unlike *M. barkeri*, showed no evidence for the formation of methanochondroitin (Fig. 2).

### Growth and physiology

All isolates produced methane from methanol and mono-, di-, and trimethylamine only in the presence of H_2_. The reaction stoichiometry for the growth on methanol is described by Eq. (1).


(1) \begin{eqnarray*}
&& {{\rm H}_{2}} + {{\rm CH}_{3}}{\rm OH} \to {{\rm CH}_{4}} + {{\rm H}_{2}{\rm O}}\\
&&\quad {\left( {\Delta {{\rm G}^{ \circ{\prime}}}} = -112.5\,\,{\rm kJ}\,{\rm mol}^{-1}\,{\rm methane} \right)}.
\end{eqnarray*}

No growth or methane formation was observed on H_2_ + CO_2_, on acetate or methanol in the absence of H_2_, or on syringate (4-hydroxy-3,5-dimethoxybenzoate), vanillate (4-hydroxy-3-methoxy-benzoate), or 3,4,5-trimethoxybenzoate in the presence of H_2_. All strains grew between 20°C and 42°C, with highest growth rates at 37°C (µ = 0.028–0.078 h^–1^; Table 1). The pH range of growth was between 6.5 and 8, with an optimum pH of 7–7.5. All strains tolerated NaCl concentrations of 300–400 mM. Growth yields ranged from 3 to 5 g dry weight per mol of methane (Table 1).

**Table 1.** Phenotypic characteristics of the new isolates compared to the type species of the genera *Methanimicrococcus* and *Methanosarcina*.

	*Methanimicrococcus*				*Methanolapillus*			*Methanosarcina*
Strain	*M. blatticola* ^#^	At1	Es2	Hf6	Ac7	Ag5	Am2	*M. barkeri* ^#^ ^§^
Isolation source	Cockroach	Cockroach	Cockroach	Cockroach	Millipede	Millipede	Millipede	Sewage sludge
Cell diameter (µm)	0.7–0.9	1.0–2.0	1.0–2.0	1.0–2.0	1.0–2.0	1.0–2.0	1.0–2.0	1.5–2.0
**Energy substrates**								
H_2_ + CO_2_	–	–	–	–	–	–	–	+
Acetate	–	–	–	–	–	–	–	+
Methanol + H_2_	+	+	+	+	+	+	+	+^b^
Methylamines + H_2_ ^a^	+	+	+	+	+	+	+	+^b^
**Growth factors** ^c^								
Acetate	+	+	+	+	+	+	+	–
Formate	–	–	s	s	–	–	+	–
Casamino acids	+	+	+	+	+	+	+	–
Yeast extract	+	–	s	–	+	+	s	–
CoM	+	+	–	–	+	+	+	–
**Other parameters**								
Doubling time (h)	3.1	23	15	19	9.5	25	20	6.9–11.5^d^
Growth yield (g/mol CH_4_)	3.5	ND	ND	5.0	ND	ND	3.5	4.6^e^
Temp. range (optimum) (°C)	20–40 (39)	25–40 (37)	25–40 (37)	25–40 (37)	25–40 (37)	25–40 (37)	25–40 (37)	(30–40)
pH range (optimum)	6.7–8.2 (7–7.5)	6.5–8 (7–7.5)	6.5–8 (7–7.5)	6.5–8 (7–7.5)	6.5–8 (7–7.5)	6.5–8 (7–7.5)	6.5–8 (7–7.5)	5–7.5 (7)
NaCl range (mM)	0–300	0–300	0–300	0–300	0–400	0–400	0–300	0–1000
Genome size (Mbp)	1.78	2.04	1.82	2.19	1.96	2.10	1.89	4.57
G + C content (mol%)	42.0	42.9	43.0	41.0	42.9	44.4	41.0	39.2

# Information from Sprenger et al. (2000, 2005).

§ Information for the type strain (Wagner 2020).

a Mono-, di-, and trimethylamine.

b Growth also in the absence of H_2_.

c Supplement is required (+), stimulatory (s), or not required (–) for growth.

d Cells grown on methanol + H_2_ (Sprenger et al. 2007).

e Cells grown on methanol + H_2_ (Müller et al. 1986).

The isolates were not inhibited by kanamycin, ampicillin, or vancomycin (100 µg/ml each). However, no growth occurred in the presence of chloramphenicol (10 µg/ml).

### Diversity and phylogenomic analysis of the intestinal clade

**Figure 3.**
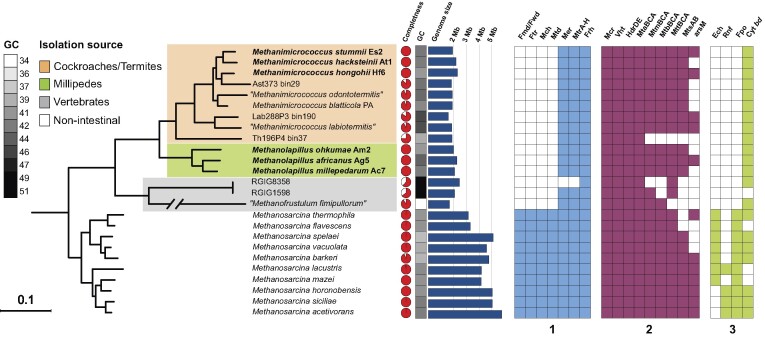
Phylogenomic tree illustrating the relationship of isolates and MAGs from animal guts to members of the genus *Methanosarcina*, and important features of their respective genomes. Newly proposed species are shown in bold. The tree is based on a concatenated alignment of 53 markers and was reconstructed with IQ-Tree under the LG+F+I+G4 model of evolution. All nodes are fully supported (>99%). The tree was rooted using other *Methanosarcinaceae* as outgroup. Genome size of MAGs was estimated based on assembly size and completeness (determined with CheckM). The presence of key genes of methanogenesis is indicated. 1, Wood–Ljungdahl pathway; 2, methyl-reducing pathway; and 3, other energy-converting membrane complexes. The multiunit complex is labeled as complete if at least half of the subunits present. For more details, see Table A3.

Phylogenomic analysis of the *Methanosarcinaceae* family, including the metagenome-assembled genomes (MAGs) obtained in previous studies (Hervé et al. 2020, Protasov et al. 2023), confirmed the monophyletic origin of the representatives from insects and millipedes (Fig. 3). The three isolates from cockroach guts fell into the radiation of the genus *Methanimicrococcus*, which comprises a single cultured representative, *M. blatticola*. The three isolates from millipedes formed a separate genus-level cluster, for which the name *Methanolapillus* is proposed (see the section “Taxonomy”). They occupy a sister position to a clade that consists of MAGs from the digestive tract of mammals and an anaerobic digester inoculated with chicken feces (Campanaro et al. 2020, Xie et al. 2021), whose members have been assigned to the genus “*Methanofrustulum*” under SeqCode (Protasov et al. 2023). Together, the three genera form a monophyletic clade that consists exclusively of methanogens from intestinal tracts.

**Figure 4.**
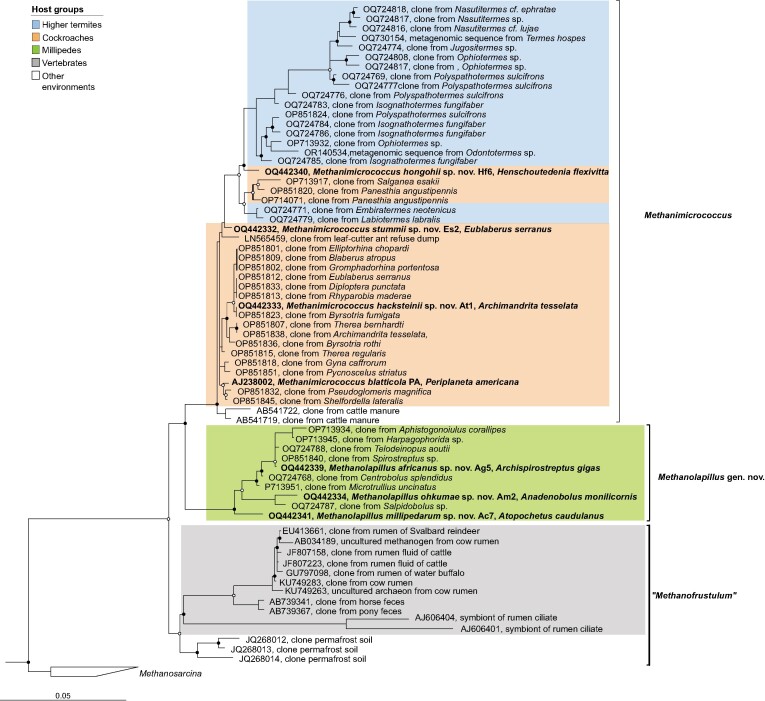
Phylogenetic 16S rRNA tree illustrating the relationship of strains isolated from arthropods to other representatives of the family *Methanosarcinaceae*. Newly proposed and type species are shown in bold. The maximum-likelihood tree is based on a curated alignment of near-full-length 16S rRNA genes (>1400 sites) and was generated using IQ-TREE under the GTR+I+G4 model of evolution. Node support was tested with ultrafast bootstrap analysis (●≥95% and ○ ≥70%; 1000 replicates). The scale bar indicates the number of substitutions per site. Color coding indicates host groups.

Also in 16S rRNA-based analyses, the strains from cockroaches fell into the radiation of the genus *Methanimicrococcus*, together with numerous uncultured representatives from a wide range of cockroaches and termites (Fig. 4). The strains from millipedes formed a separate cluster that consists exclusively of sequences derived from diverse millipede species that were assigned to the genus *Methanolapillus* (Protasov et al. 2023). Both clusters occupy a sister position to uncultured methanogens from the digestive tract of various mammals, including a few clones from permafrost soil that may have originated from animal feces. Members of the genus “Methanofrustulum” were first detected in the cow rumen (Tajima et al. 2001, Wright et al. 2007) and subsequently in reindeer (Sundset et al. 2009), water buffalo (Chaudhary et al. 2012), yak (Huang et al. 2012), and sheep (Huang et al. 2016), and also in horse feces (Murru et al. 2018). The low abundance of “*Methanofrustulum*” in mammalian samples stands in sharp contrast to the situation in arthropod guts, where *Methanimicrococcus* or *Methanolapillus* may represent up to 97% of the archaeal community (Protasov et al. 2023).

The closest cultured relatives of intestinal *Methanosarcinales* are members of the genus *Methanosarcina*, which occur in soil, sediments, or anaerobic digesters (Wagner 2020). The low 16S rRNA gene sequence similarities (94.4%–96.0%) between the strains from millipedes (Ac7, Ag5, and Am2) and representatives of the genus *Methanimicrococcus* and the average nucleotide identities of their genomes (ANI, 76%–78%) (Fig. A2) support their placement in a separate genus (Yarza et al. 2014, Jain et al. 2018).

### Energy metabolism

The members of the intestinal clade have estimated genome sizes ranging between 1.58 and 2.24 Mb, which is significantly smaller than the values of their closest relatives in the genus *Methanosarcina* (Table 1; Fig. 3; Fig. A3).

**Figure 5.**
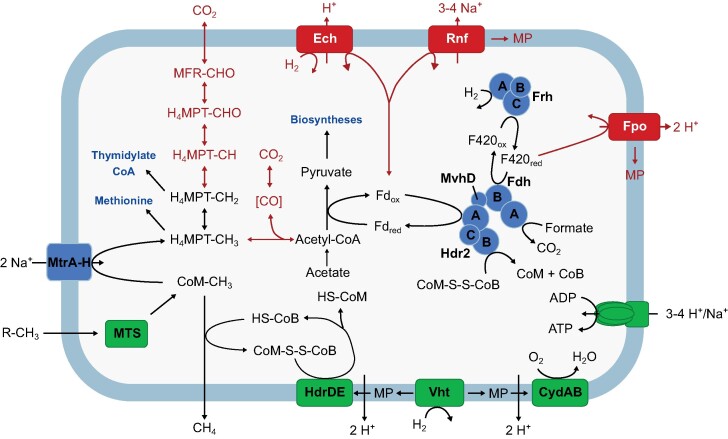
Metabolic pathways of methyl-reducing *Methanosarcinales*. Reactions and enzyme complexes that are absent from all members of the intestinal clade are shown in red. Enzyme complexes involved in energy metabolism are shown in green, those involved in anabolism are shown in blue. Scheme based on information in Fig. 3 and Table A3. MTS, methyl transferase systems; MP, methanophenazine.

Comparative genome analysis revealed that in all cases, the reduction in genome size is accompanied by a loss of both catabolic and anabolic pathways. The most striking feature of the intestinal lineages is the absence of the Wood–Ljungdahl pathway and other prominent elements of the energy metabolism of their next relatives in the genus *Methanosarcina*. This documents that the severe genome reduction previously observed in *M. blatticola* is a common trend among all members of the entire intestinal clade and most likely reflects features that were present already in the last common ancestor of the three animal-associated lineages (Thomas et al. 2021) (Fig. 5).

The breakdown of the Wood–Ljungdahl pathway in all members of the intestinal clade explains their inability to reduce CO_2_ for methane production, which had already been demonstrated for *M. blatticola* (Sprenger et al. 2005). The conservation of methyl-tetrahydromethanopterin (H_4_MPT): CoM methyl transferase (MtrA–H), and methylene-H_4_MPT reductase (Mer) in all genomes is most likely due to their role in anabolism, providing methyl-H_4_MPT and methylene-H_4_MPT as precursors for the biosynthesis of acetyl-CoA, methionine, thymidylate, and coenzyme A (Thauer 2012). Notably, methenyl-H_4_MPT cyclohydrolase (Mch) is absent from all members of the intestinal clade. The absence of CdhA, CdhB (and CdhC in case of *Methanolapillus*), which are key components of the CO dehydrogenase/acetyl-CoA synthase (CODH/ACS) complex and essential for autotrophic growth, explains the acetate requirement of all strains from the intestinal clade (Table 1).

All members of the intestinal clade lack the energy-conserving membrane-bound complexes typical of other *Methanosarcinales*, such as F_420_H_2_ dehydrogenase (Fpo), Rnf complex, and an energy-converting hydrogenase (Ech). An MtrA–H complex is present but it likely serves only in the methylation of H_4_MPT for anabolic purposes. The only means for energy conservation during H_2_-dependent reduction of methyl-CoM is the short electron-transport chain formed by the methanophenazine-reducing [NiFe] hydrogenase (VhtACG) and the methanophenazine-oxidizing heterodisulfide reductase (HdrDE) (Fig. 5). All members of the intestinal clade possess several methyltransferase systems that transfer the methyl groups of methanol (Mta) and mono-, di-, and trimethylamine (Mtm, Mtb, and Mtt) to CoM for their subsequent reduction to methane (Fig. 3). They are organized in gene clusters that comprise a substrate-specific component, a methyl-accepting corrinoid protein (CoP), and in most cases also homologs of the second methyltransferase that transfers the methyl group from CoP to CoM (for details, see Table A3). In addition, all isolates encode homologs of a methylthiol: CoM methyltransferase (Mts), which is involved in methanogenesis from dimethylsulfide and methylmercaptopropionate in *M. barkeri* (Tallant et al. 2001). In several *Methanimicrococcus* and *Methanolapillus* genomes, we found homologs of an arsenite methyltransferase (ArsM). Homologs of the recently discovered methyltransferase of *Methermicoccus shengliensis*, which transfers methyl groups from methoxylated aromatic compounds (Kurth et al. 2021), were not detected, which is consistent with the inability of the isolates to utilize such substrates.

Many members of the genus *Methanosarcina* and other *Methanosarcinales* use glycogen and polyphosphates as storage compounds (Ferry 2012, Wang et al. 2019). Genes involved in glycogen biosynthesis were absent from all representatives of the intestinal clade, whereas the pathways for polyphosphate synthesis and degradation were present (Table A3).

### Anabolism

Comparative genome analysis revealed that most of the intestinal *Methanosarcinales* lack the biosynthetic pathways for aromatic amino acids phenylalanine, tyrosine, and tryptophan with some *Methanimicrococcus* likely also not able to synthesize valine, leucine, and isoleucine (Table A3). In contrast to members of the genus *Methanosarcina*, which are typically capable of dinitrogen fixation, none of the genomes of the intestinal clade encoded homologs of nitrogenase (NifDHK).

All members of the intestinal clade lack the biosynthetic pathway for methanofuran (Table A3) (Wang et al. 2015). Only *Methanimicrococcus* strains Es2 and Hf6 retained the l-phosphoserine-dependent pathway for the biosynthesis of CoM, which is common among *Methanosarcinales* (Graham et al. 2009). The absence of the pathway from other *Methanimicrococcus* spp., including *M. blatticola*, and all members of the genus *Methanolapillus*, explains their requirement for CoM as growth factor (Table 1). Genes encoding the PEP-dependent pathway or the bacterial pathway for CoM biosynthesis were not found.

While most genomes encode the complete biosynthetic pathways for pantothenate, niacinamide, and thiamine, the pathways for the synthesis of several other cofactors are absent or incomplete (Table A3). Most isolates should have a requirement for folate and (except for the genus *Methanolapillus*) pyridoxalphosphate. The pathway for biotin synthesis is absent but compensated by an ABC transporter for biotin. Thiamine cannot be synthesized *de novo* but via a salvage pathway requiring some precursors from the environment (Jenkins et al. 2007). Riboflavin biosynthesis lacks four of the canonical enzymes, but since the same enzymes are absent also in *Methanosarcina* spp. that grow on mineral medium, it is likely that the pathway for riboflavin biosynthesis in *Methanosarcinales* differs from the canonical pathway.

The pathways for the biosynthesis of tetrapyrroles are fully preserved in all genomes of the intestinal clade (Table A3). They comprise both the initial steps common to all tetrapyrroles and the upper parts of the pathways leading to cofactor F_430_, cobalamine/cobamides and heme (Bryant et al. 2020).

### Cell envelope and reactive oxygen species

All genomes encode homologs of the protein MA0829, which self-assembles into the two-dimensional crystalline array forming the S-layer of *Methanosarcina acetivorans* (Francoleon et al. 2009, Arbing et al. 2012). Although the identity scores are rather low (28%–36%; Table A3), this is consistent with the apparent presence of an S-layer in the electron micrographs of *Methanimicrococcus* and *Methanolapillus* spp. (Fig. 2).

The nonmotile phenotype of the isolates is consistent with the absence of the archaellum operon (Arl) and the chemotaxis operon (Che) present in some *Methanosarcina* (Jarrell et al. 2021) from all members of the intestinal clade. Homologs of the subcluster ArlIJ, which were detected in a few genomes, are likely involved in protein export, as predicted already for *M. mazei* (Deppenmeier et al. 2002).

All members of the intestinal clade encode catalase, thioredoxin-dependent peroxiredoxins, and superoxide reductase. While Cu–Fe superoxide dismutase and F_420_H_2_ oxidase are absent, a Fe–Mn superoxide dismutase (SodA) was detected in members of the genus *Methanimicrococcus* (Table A3). Notably, the homolog of cytochrome *bd* (CydAB), which is encoded by many members of the genus *Methanosarcina* (Baughn and Malamy 2004, Brochier-Armanet et al. 2009), is conserved in all members of the intestinal clade (Fig. 3). Phylogenetic analysis revealed a close relationship to the high-affinity quinol oxidases of *Bacillota* (Fig. 7).

## Discussion

### Genome reduction in the intestinal clade

**Figure 6.**
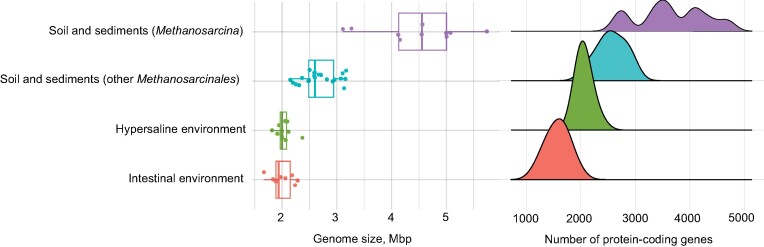
Comparison of genome size and number of protein-coding genes in the genus *Methanosarcina* and other *Methanosarcinales* from different environments. Soil and sediments: *Methanococcoides, Methanolobus*, and *Methanomethylovorans*. Hypersaline environments: *Methanohalobium, Methanohalophilus*, and *Methanosalsum*. Intestinal environments: *Methanimicrococcus, Methanolapillus*, and “*Methanofrustulum*”. For details, see Tables A1 and A2.

While members of the genus *Methanosarcina* have experienced a massive genome expansion due to horizontal gene transfer from bacteria and possess the largest genomes in the archaeal domain (Deppenmeier et al. 2002, Garushyants et al. 2015), members of the intestinal clade (genera *Methanimicrococcus, Methanolapillus*, and “*Methanofrustulum*”) show a significant genome reduction (Fig. 6). Small genomes with an average size of 2.0 Mb are also found among *Methanosarcinaceae* from hypersaline environments (genera *Methanohalobium, Methanohalophilus*, and *Methanosalsum*) (Oren 2014a). While genome reduction in intestinal microorganisms has been explained with the loss of nonessential genes in nutrient-rich environments (Morris et al. 2012), genome streamlining in hypersaline habitats may be attributed to a strong purifying selection driven by the extremely nutrient-poor environment (Wolf and Koonin 2013, Giovannoni et al. 2014). Notably, the average number of protein-encoding genes and the corresponding average coding density in the intestinal clade of *Methanosarcinales* is significantly lower than in those from hypersaline environments (Fig. 6; Figure A4), suggesting that numerous genes were pseudogenized but not yet eliminated from the genome due to the absence of a strong purifying selection (Koonin and Wolf 2008). This underscores that the environment is an important driver of genome evolution in *Methanosarcinales*.

The loss of the Wood–Ljungdahl pathway and the absence of many energy-converting membrane complexes present in the genus *Methanosarcina* (Fpo, Ech, and Rnf) is common to all members of the intestinal clade (Fig. 3). As a consequence, members of the intestinal clade can conserve energy only via the methanophenazine-linked electron transport chain that is formed by the complexes VhtACG and HdrDE (Abken et al. 1998). Moreover, in the absence of Ech and Rnf, the reduced ferredoxin required for anabolism must be provided by other means.

A MvhAGD [NiFe] hydrogenase is generally absent from the order *Methanosarcinales* (Mand and Metcalf 2019), precluding the formation of reduced ferredoxin via a cytosolic MvhAGD–HdrABC complex. Since members of the intestinal clade possess both formate dehydrogenase (FdhAB) and an MvhD homolog fused with HdrABC, an alternative route would be the reduction of ferredoxin with formate, using an FdhAB–MvhD–HdrABC complex, as shown for *Methanococcus maripaludis* (*Methanococcales*) and *Methanoculleus thermophilus* (*Methanomicrobiales*) (Costa et al. 2010, Milton et al. 2018, Abdul Halim et al. 2021). An alternative electron donor for this complex would be F_420_H_2_, as suggested already by Thomas et al. (2021) for *M. blatticola*. The cytoplasmic HdrABC present in members of the intestinal clade belongs to the same subclade as its homolog in *M. acetivorans* (HdrA2B2C2), which couples F_420_H_2_ oxidation to the simultaneous reduction of ferredoxin and CoM-S-S-CoB via flavin-based electron bifurcation (Yan et al. 2017). A structural and mechanistic analysis of the FdhAB–MvhD–HdrABC complex in *Methanospirillum hungatei* (*Methanomicrobiales*) revealed that it uses both formate and F_420_H_2_ as electron donor (Watanabe et al. 2021). Since we observed a growth requirement for formate only in strain Am2, it is likely that members of the intestinal clade do not use formate for ferredoxin reduction but instead use F_420_H_2_ generated by the F_420_-reducing [NiFe] hydrogenase (Frh) (Fig. 5).

While MtrA–H is the only site of energy conservation in hydrogenotrophic, CO_2_-reducing methanogens, members of *Methanosarcinales* possess additional ion-translocating membrane-bound complexes, which increases the ATP yield per mol of methane and explains why their growth yields are the highest among methanogens (Thauer et al. 2008, Mand and Metcalf 2019). Growth yields among members of the intestinal clade (3.0–5.0 g/mol methane; Table 1) are in the same range as those of *M. barkeri* grown on methanol + H_2_ with acetate as carbon source (4.6 g/mol methane) (Müller et al. 1986) but substantially lower than those of *M. barkeri* grown only on methanol (7.2 g/mol) (Weimer and Zeikus 1978). A similar growth yield (4 g/mol methane) was reported for the obligately methyl-reducing *Methanosphaera stadtmanae* (Miller and Wolin 1985), which oxidizes the reduced Fd produced by HdrABC with an energy-converting hydrogenase (Ehb (Thauer et al. 2008)). In this context, it is important to note that the free-energy changes of the methyl-reducing pathway (Eq. 1) and the methyl-disproportionating pathway (Eq. 2) are almost identical under standard conditions, whereas the hydrogen-dependent reduction of methanol to methane becomes energetically less favorable at lower hydrogen partial pressure (Thauer et al. 2008, Feldewert et al. 2020).


(2) \begin{eqnarray*}
&& {4\,{\rm CH}_{3}{\rm OH}} \to {3\,{\rm CH}_{4}} + {{\rm CO}_{2}} + {2{\rm H}_{2}{\rm O}}\\
&&\quad {\left( {\Delta {{\rm G}^{ \circ{\prime}}} = -106.5\,{\rm kJ}\,{\rm mol}^{- 1}\,{\rm methane}} \right)}.
\end{eqnarray*}

The lower growth yield of the obligately methyl-reducing *Methanomassiliicoccus luminyensis* (2.4 g/mol methane) (Kröninger et al. 2017) is explained by the unique energy metabolism of *Methanomassiliicoccales*, where only every second CoM-S-S-CoB is reduced via an energy-converting membrane-bound complex (Lang et al. 2015, Kröninger et al. 2019).

### Evolution of the methylotrophic metabolism

The intestinal *Methanosarcinales* clade represents a case of obligate H_2_-dependent methylotrophy. They utilize a short electron transport chain that consists of only membrane-bound heterodisulfide reductase HdrDE and membrane-bound VhtACG hydrogenase. The intestinal clade is embedded among *Methanosarcinales* that have the capacity for hydrogenotrophic, methyl-disproportionating, and aceticlastic methanogenesis. The transition to obligate H_2_-dependent methyl-reducing methanogenesis was likely triggered by the dispersal of an ancestral lineage of *Methanosarcinales* into the H_2_-rich gut environment. Since methanol oxidation in *M. barkeri* is inhibited in the presence of H_2_ (Mand et al. 2018), it is possible that this restricted the intestinal clade to using only the methyl-reducing pathway. Once the selective pressure on methyl oxidation had been removed, members of the clade lost the Wood–Ljungdahl pathway and the membrane-bound complexes involved in methyl oxidation and became obligately H_2_-dependent methylotrophs. The loss of the Wood–Ljungdahl pathway also lead members of the clade to rely on the gut environment for precursors of biosynthesis. That would also explain why intestinal *Methanosarcinales* are rarely detected in nongut environments (Thomas et al. 2022). The oligotrophic conditions in sediments and soils, where other *Methanosarcinales* are typically encountered, does not meet the growths requirement of the auxotrophic intestinal species. Rare observations of *Methanimicrococcus*-related sequences in soil (see Fig. 4) may indicate fecal contaminations from mammals and/or arthropods.

Biochemically characterized species of obligate methyl-reducing methanogens (*M. stadtmanae, M. luminyensis, Methanonatronarchaeum thermophilum*, and *M. blatticola*) use similar pathways to channel methyl groups to a methyl-CoM reductase (Mcr) via various methyltransferase systems (Sprenger et al. 2005, Thauer et al. 2008, Kröninger et al. 2017, Steiniger et al. 2022). However, they all differ in the way they produce an ion gradient for ATP production and do not use the MtrA-H complex that is common for other methanogens (summarized in Garcia et al. 2022). The differences in the pathways indicate an independent origin of methylotrophy in these lineages. Moreover, it has been suggested that also several other lineages of methanogens are obligately methylotrophic, based either on comparative genome analysis alone (Nobu et al. 2016, Vanwonterghem et al. 2016) or in combination with a physiological characterization of isolates and enrichment cultures (Kohtz et al. 2024, Krukenberg et al. 2024, Lynes et al. 2024, Wu et al. 2024). The differences between these lineages suggest a convergent evolution of the obligate methyl-reducing pathways, starting with a unique biochemical basis in different lineages. Evidently, the gut environment stands out as a significant hub for obligate methyl-reducing methanogens harboring representatives such as *Methanosphaera* spp., *Methanomethylophilaceae*, and intestinal *Methanosarcinales*. This implies that the gut environment not only supports but potentially even facilitates the transition to obligate H_2_-dependent methyl-reducing methanogenesis, as shown for intestinal *Methanosarcinales* in the present study.

### The loss of biosynthetic capacities in the intestinal clade

All strains of intestinal *Methanosarcinales* have pronounced auxotrophic phenotypes, requiring growth factors such as acetate, formate, and/or other, so far unidentified components of yeast extract. A requirement for CoM is common not only among representatives of the genera *Methanimicrococcus* and *Methanolapillus* (Sprenger et al. 2000, Table 1) but also among gut-associated lineages of *Methanobacteriales* (*Methanobrevibacter* spp., *M. stadtmanae*) and *Methanomassiliicoccales* (*Ca*. Methanoplasma termitum) (Oren 2014b, Lang et al. 2015). In arthropod guts, members of the intestinal clade always co-occur with other methanogens (Protasov et al. 2023), suggesting a dependency of these species on other, CoM-producing methanogens in the intestinal community.

The absence of a functional CODH/ACS complex and presence of an acetate transporter (Welte et al. 2014, Ribas et al. 2019) explains the acetate requirement of all isolates in the genera *Methanimicrococcus* and *Methanolapillus* and predicts the same also for all uncultured members of the intestinal clade. It is intriguing that *Methanimicrococcus* and *Methanolapillus* differ in the number of Cdh subunits; *Methanimicrococcus* have only two out of five subunits, while *Methanimicrococcus* possess three subunits (Table A3). Like CoM auxotrophy, also a growth requirement for acetate is common among methanogens isolated from intestinal environments, such as *Methanobrevibacter* or *Methanosphaera* spp. (Oren 2014b). It is likely that the loss of acetate production via the CODH/ACS complex is promoted by the high acetate concentration in intestinal environments.

Tetrapyrroles are essential components of many enzymes and cofactors of methanogens, such as cofactor F_430_ (the prosthetic group of methyl-CoM reductase), cobalamin/cobamide (the prosthetic group of methyltransferases), and heme (the prosthetic group of cytochromes) (Matthews et al. 2008, Bryant et al. 2020). While cofactor F_430_ is unique to methanogens and is synthesized by all species isolated to date, cobalamin/cobamide are produced by many bacteria and can be scavenged from the environment (Sokolovskaya et al. 2020). The presence of the complete pathway for the synthesis of cobalamin and the lack of a B_12_ transporter in all isolates of the intestinal clade (Table A3) indicates the importance of B_12_-dependent methyltransferases in their energy metabolism. Also, the biosynthetic pathway for pyrrolysine, an essential amino acid in methylamine methyltransferases, in almost all genomes (Table A3) is in agreement with the ability to grow on methylamines (Table 1) (Rother and Krzycki 2010).

Heme can be synthesized by all methanogens that produce cytochromes (*Methanosarcinales* and *Methanonatronarchaeales*; Mand and Metcalf 2019, Steiniger et al. 2022) and possibly a few uncultured lineages (Ou et al. 2022). Like other archaea, *Methanosarcinales* synthesize heme via the siroheme pathway (Dailey et al. 2017). The presence of the pathway in all members of the intestinal clade and the absence of a heme transporter reflects the importance of cytochrome-containing complexes (VhtACG and HdrDE) in their energy metabolism.

Amino acid and vitamin auxotrophies are common for complex microbial communities such as gut microbiota (Zengler and Zaramela 2018, Ramoneda et al. 2023). While many *Methanosarcina* species from soils and sediments grow on medium without organic growth factors (Wagner 2020), members of the intestinal clade are auxotrophic for several amino acids and vitamins (Table A3). Again, the same applies to other host-associated archaea, such as *Methanobrevibacter* spp. (Leadbetter and Breznak 1996) and *M. stadtmanae* (Miller and Wolin 1985).

The intestinal clade members do not possess the genes for the synthesis and activation of *N*-acetyl-galactosamine and glucuronic acid, the precursors for methanochondroitin (Hartmann and König 1991), as already shown for *M. blatticola* (Thomas et al. 2021). This is consistent with absence of aggregates in all isolates from the intestinal clade under tested growth conditions and the apparent absence of methanochondroitin from their cell envelope (Fig. 2). However, like most methanogens, members of *Methanosarcinales* possess a proteinaceous S-layer that is linked to the cytoplasmic membrane (van Wolferen et al. 2022). Homologs of the S-layer protein were present in all members of the intestinal clade, and an S-layer was visualized in two of the isolates.

Although members of the genus *Methanosarcina* are typically immotile, *M. acetivorans* possesses genes required for the synthesis of an archaeal flagellum (archaellum) and chemotaxis (Wagner 2020). All isolates of the genera *Methanimicrococcus* and *Methanolapillus* are immotile and lack the genes for an archaellum or chemotaxis. Epifluorescence microscopy of the gut wall of cockroaches showed cells resembling *Methanimicrococcus* spp. attached to the inner surface of the gut epithelium (Sprenger et al. 2000). It is likely that an attachment to intestinal surfaces is a general strategy for all members of the intestinal clade and led to a loss of both motility and chemical sensing upon transition to the gut environment, as already suggested for *M. blatticola* (Thomas et al. 2021).

### Relationship to oxygen

Although oxygen penetrates into the hindgut of arthropods and renders the gut periphery a microoxic habitat, the inner surface of the hindgut wall is often colonized by methanogens (Brune 2019). It is long known that both *Methanobrevibacter* and *Methanisarcina* species are more oxygen tolerant than other methanogens (Kiener and Leisinger 1983). Experiments with agar-gradient tubes and dense cell suspensions documented that arthropod-derived members of the genus *Methanobrevibacter* and *M. blatticola* remove O_2_ from their environment (Leadbetter and Breznak 1996, Sprenger et al. 2007, Tholen et al. 2007). *Methanobrevibacter* species reduce O_2_ to water via an F_420_H_2_ oxidase (FprA) (Seedorf et al. 2004). Like other *Methanosarcinales*, members of the intestinal clade encode enzymes for the detoxification of reactive oxygen species, such as catalase (Shima et al. 1999), superoxide dismutase (Brioukhanov et al. 2000), and superoxide reductase (Krätzer et al. 2011), and a thioredoxin system (McCarver and Lessner 2014, Kumar et al. 2015). Homologs of FprA, however, are absent (Table A3).

**Figure 7.**
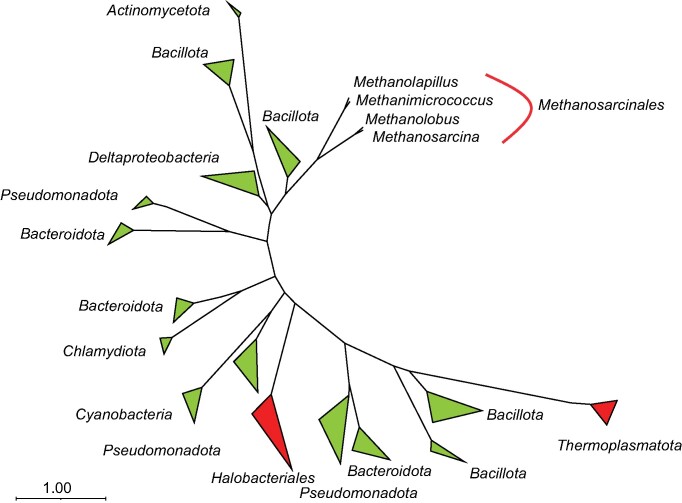
Unrooted phylogenetic tree of prokaryotic cytochrome *bd* (CydA). Homologs from archaea are marked in red, those from bacteria are marked in green. All nodes are highly supported (98%–100%, ultrafast bootstrap analysis).

A plausible candidate for O_2_ removal in the intestinal clade is cytochrome *bd* (CydAB), which is present in the genomes of many *Methanosarcinaceae* and is most closely related to the high-affinity quinol oxidases of *Bacillota* (Fig. 7). Homologs of this enzyme allow “obligate anaerobes” such as *Bacteroides fragilis* or *Desulfovibrio gigas* to remove O_2_ from their immediate environment and even support a respiratory energy metabolism under microoxic conditions, which gave rise to the concept of “nanaerobes” (Lemos et al. 2001, Baughn and Malamy 2004). We propose that the CydAB of *Methanosarcinales* is part of a methanophenazine-linked respiratory chain (Fig. 5). Analogous to the hydrogen-dependent reduction of heterodisulfide via VhtACG–methanophenazine–HdrDE, which produces an electrochemical proton gradient during methanogenesis via a mechanism similar to that of the bacterial quinone loop (Deppenmeier 2004), also the hydrogen-dependent reduction of O_2_ via VhtACG–methanophenazine–CydAB may serve to conserve energy by electron transport phosphorylation.

### Taxonomy

Recently, several members of the intestinal *Methanosarcinales*, including the isolates in the present study, have been described as new species under SeqCode, using their genomes as type material (Protasov et al. 2023). However, taxa described under SeqCode are not considered validly published under the ICNP (Göker et al. 2022) because the latter generally requires axenic cultures to be deposited as type material in two culture collections. Here, we provide a formal description of the six isolates and the new genus *Methanolapillus* under the ICNP, following also minimal standards for the description of methanogens (Prakash et al. 2023).

### *Methanolapillus* gen. nov. Protasov and Brune

Etymology: Me.tha.no.la'pil.lus. N.L. neut. N. *methanum*, methane; N.L. pref. *methano-*, pertaining to methane; L. masc. n. *lapillus*, a pebble, gem, jewel; N.L. masc. n. *Methanolapillus*, a methane-producing jewel.

Description: Cells are irregular cocci with a diameter of 1.0–2.0 µm, occurring singly or sometimes in small clusters. Form lens-shaped, cream-colored colonies in deep-agar cultures. Nonmotile. Obligately hydrogen-dependent methyl-reducing methanogens; reduce methanol and methylamines, but not methoxylated aromatic compounds. No methane formation from formate, acetate, or ethanol. Mesophilic. Require complex medium with acetate and Casamino acids or peptone. Yeast extract and formate may be stimulatory or essential for growth.

Type species: *Methanolapillus millepedarum* sp. nov.

### *Methanolapillus millepedarum* sp. nov. Protasov and Brune

Etymology: mil.li.pe.da'rum. L. fem. n. *millepeda*, a millipede; gen. pl. n. *millepedarum*, of millipedes.

Description: Cells are irregular cocci with a diameter of 1.0–2.0 µm that occur singly or in small clusters. Growth optimum at 37°C (range 20°C–42°C); no growth at 15°C or 45°C. Optimal pH is 7.0–7.5 (range 6.5–8). NaCl optimum is 100 mM (range 0–400 mM). Requires acetate, yeast extract, Casamino acids, and CoM. The type strain was isolated from the hindgut of the millipede *A. caudulanus*.

Type strain: Ac7^T^ = DSM 114425^T^ = JCM 39380^T^.

Genome: GCA_032594115; OQ442341 (16S rRNA gene).

### *Methanolapillus africanus* sp. nov. Protasov and Brune

Etymology: af.ri.ca'nus, L. masc. adj. *africanus*, African; referring to the geographic origin of the host.

Description: Cells are irregular cocci with a diameter of 1.0–2.0 µm that occur singly or in small clusters. Growth optimum at 37°C (range 20°C–42°C); no growth at 15°C or 45°C. Optimal pH is 7.0–7.5 (range 6.5–8). NaCl optimum is 100 mM (range 0–400 mM). Requires acetate, yeast extract, Casamino acids, and CoM. The type strain was isolated from the hindgut of the millipede *A. gigas*.

Type strain: Ag5^T^ = DSM 115569^T^ = JCM 39381^T^.

Genome: GCA_032714475; OQ442339 (16S rRNA gene).

### *Methanolapillus ohkumae* sp. nov. Protasov and Brune

Etymology: oh.ku'mae. N.L. gen. masc. n. *ohkumae*, of Ohkuma, named after Moriya Ohkuma in recognition of his important contributions to arthropod gut microbiology.

Description: Cells are irregular cocci with a diameter of 1.0–2.0 µm that occur singly or in small clusters. Growth optimum at 37°C (range 20°C–42°C); no growth at 15°C or 45°C. Optimal pH is 7.0–7.5 (range 6.5–8). NaCl optimum is 100 mM (range, 0–300 mM). Requires acetate, formate, and CoM; yeast extract is stimulatory. The type strain was isolated from the hindgut of the millipede *A. monilicornis*.

Type strain: Am2^T^ = DSM 114424^T^ = JCM 39382^T^.

Genome: GCA_032594355; OQ442334 (16S rRNA gene).

### *Methanimicrococcus* corrig. Sprenger et al. (2000) emend

Etymology: Me.tha.ni.mi.cro.coc'cus. N.L. neut. N. *methanum*, methane; Gr. masc. adj. *mikros*, small; N.L. masc. n. *coccus*, a spherical microbe; N.L. masc. n. *Methanimicrococcus* a small methane-forming coccus.

Description: Cells are irregular cocci with a diameter of 0.7–2.0 µm, occur singly or in small clusters. Form lens-shaped, cream-colored colonies in deep-agar cultures. Nonmotile. Obligately hydrogen-dependent methyl-reducing methanogens; reduce methanol and methylamines, but not methoxylated aromatic compounds. No methane formation from formate, acetate, and ethanol. Mesophilic. Require complex medium with acetate and Casamino acids (Casamino acids can be substituted by peptone or tryptic soy broth). Yeast extract and formate may be stimulatory or essential for growth.

Type species: *M. blatticola* Sprenger et al. (2000).

### *Methanimicrococcus hacksteinii* sp. nov. Protasov and Brune

Etymology: hack.stein'i.i. N.L. gen. masc. n. *hacksteinii*, named after Johannes H.P. Hackstein in recognition of his important contributions to arthropod gut microbiology.

Description: Cells are irregular cocci with a diameter of 1.0–2.0 µm that occur singly or in small clusters. Growth optimum at 37°C (range 20°C–42°C); no growth at 15°C or 45°C. Optimal pH is 7.0–7.5 (range 6.5–8). NaCl optimum is 100 mM (range, 0–300 mM). Requires acetate, Casamino acids, and CoM. The type strain was isolated from the hindgut of the cockroach *A. tessellata*.

Type strain: At1^T^ = DSM 115570^T^ = JCM 39383^T^.

Genome: GCA_032714515; OQ442333 (16S rRNA gene).

### *Methanimicrococcus stummii* sp. nov. Protasov and Brune

Etymology: stumm'i.i. N.L. gen. masc. n. *stummii*, of Stumm, named in honor of Claudius K. Stumm for his important contributions on the symbiosis of methanogens with anaerobic protists.

Description: Cells are irregular cocci with a diameter of 1.0–2.0 µm that occur singly or in small clusters. Growth optimum at 37°C (range 20°C–42°C); no growth at 15°C or 45°C. Optimal pH is 7.0–7.5 (range 6.5–8). NaCl optimum is 100 mM (range 0–300 mM). Requires acetate and Casamino acids; formate and yeast extract are stimulatory. The type strain was isolated from the hindgut of the cockroach *E. serranus*.

Type strain: Es2^T^ = DSM 114387^T^ = JCM 39384^T^.

Genome: GCA_032594435; OQ442332 (16S rRNA gene).

### *Methanimicrococcus hongohii* sp. nov. Protasov and Brune

Etymology: hon.goh'i.i. N.L. gen. masc. n. *hongohii*, of Hongoh, named after Yuichi Hongoh in recognition of his important contributions to arthropod gut microbiology.

Description: Cells are irregular cocci with a diameter of 1.0–2.0 µm that occur singly or in small clusters. Growth optimum at 37°C (range 20°C–42°C); no growth at 15°C or 45°C. Optimal pH is 7.0–7.5 (range 6.5–8). NaCl optimum is 100 mM (range 0–300 mM). Requires acetate and Casamino acids; formate is stimulatory. The type strain was isolated from the hindgut of the cockroach *H. flexivitta*.

Type strain: Hf6^T^ = DSM 114388^T^ = JCM 39385^T^.

Genome: GCA_032594095; OQ442340 (16S rRNA gene).

## Supplementary Material

fiae111_Supplemental_Files
