# Supplementary material for: Genome reduction in novel, obligately methyl-reducing Methanosarcinales isolated from arthropod guts (Methanolapillus gen. nov. and Methanimicrococcus)
Source: FEMS Microbiol Ecol. 2024 Aug 6;100(9):fiae111. doi: 10.1093/femsec/fiae111 (PMC11362671; doi:10.1093/femsec/fiae111)
Supplement: fiae111_Supplemental_Files [file fiae111_supplemental_files.zip › supp data Figures_A1-A4.pdf]

Supplementary Figures

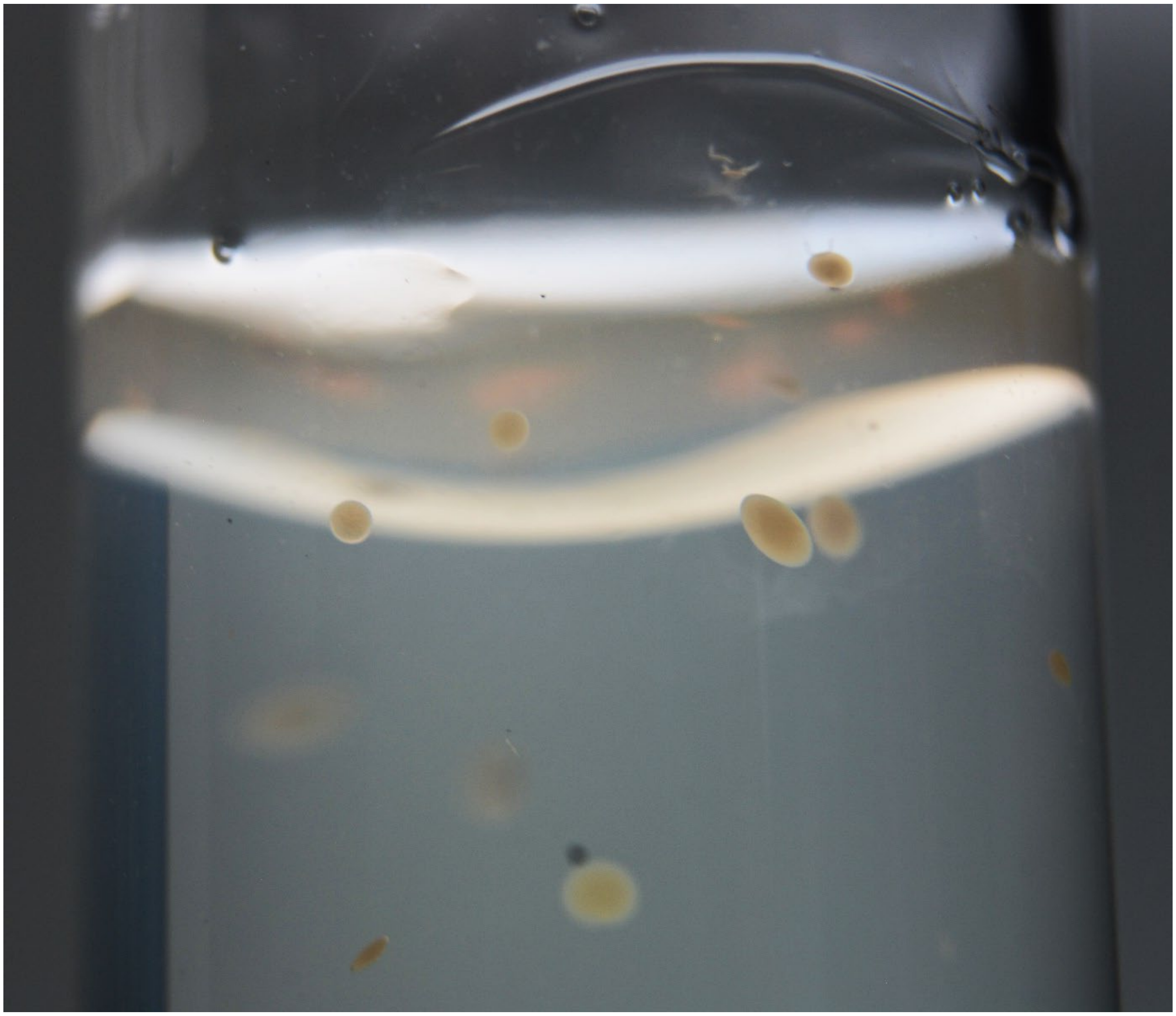

Figure A1. Colonies of *Methanolapillus africanus* Ag5 in a deep-agar dilution tube, one month after inoculation.

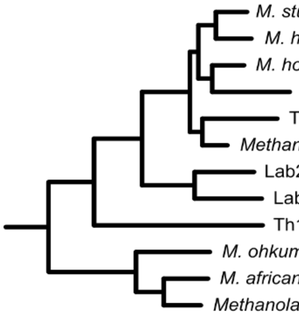

	Es2	At1	Hf6	Ast373 bin29	TD116 bin10	*M. blatticola*	Lab288P3 bin190	Lab288P3 bin112	Th196P4 bin37	Am2	Ag5	Ac7
M. stummii Es2	-	85	82.8	80.1	80.4	80.4	78.5	78.7	<78	<78	<78	<78
M. hacksteinii At1	97.3	-	81.7	80.4	80.1	80.1	78.6	78.7	<78	<78	<78	<78
M. hongohii Hf6	97.3	97.3	-	80.4	80.1.	80.1	78.3	78.3	<78	<78	<78	<78
Ast373 bin29	NA	NA	NA	-	79.5	80.6	78.1	78.1	<78	<78	<78	<78
TD116 bin10	NA	NA	NA	NA	-	82.4	78.3	78.4	<78	<78	<78	<78
Methanimicrococcus blatticola PA	97.3	97.3	97.3	NA	NA	-	78.5	78.4	<78	<78	<78	<78
Lab288P3 bin190	NA	NA	NA	NA	NA	NA	-	<78	<78	<78	<78	<78
Lab288P3 bin112	NA	NA	NA	NA	NA	NA	NA	-	<78	<78	<78	<78
Th196P4 bin37	NA	NA	NA	NA	NA	NA	NA	NA	-	<78	<78	<78
M. ohkumae Am2	95.3	95.2	95.2	NA	NA	94.5	NA	NA	NA	-	79	79.5
M. africanus Ag5	95.7	95.3	95.1	NA	NA	94.4	NA	NA	NA	97.3	-	82.5
Methanolapillus millepedarum Ac7	96	95.9	95.6	NA	NA	94.9	NA	NA	NA	97	98.2	-

ANI (%)

16S rRNA gene sequence identity (%)

Figure A2. Pairwise comparison of 16S rRNA gene sequence identity and average nucleotide identity (ANI) between members of the genera *Methanimicrococcus* and *Methanolapillus*. The background colors of the cells indicate values at genus range. ANI values < 78% are below the cutoff of the FASTAni tool. NA, no 16S rRNA gene recovered in the MAG.

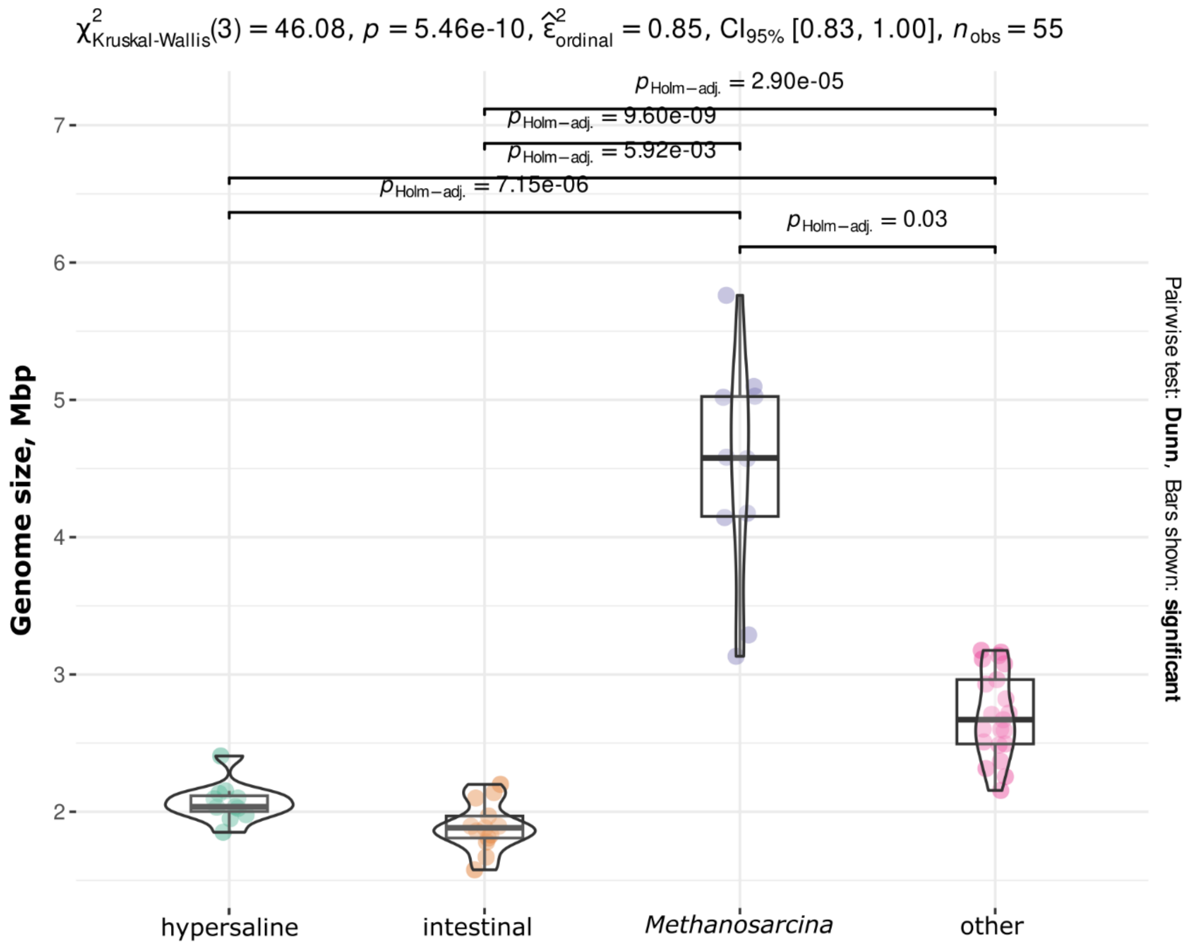

Figure A3. Comparison of genome size between different groups of *Methanosarcinales* (same dataset as in Fig. 6). Genome sizes were not normally distributed (Shapiro-Wilk normality test, $p < 0.05$). Significant differences between the mean values are indicated (Kruskal–Wallis test and post-hoc Dunn tests with Holm p-value adjustment).

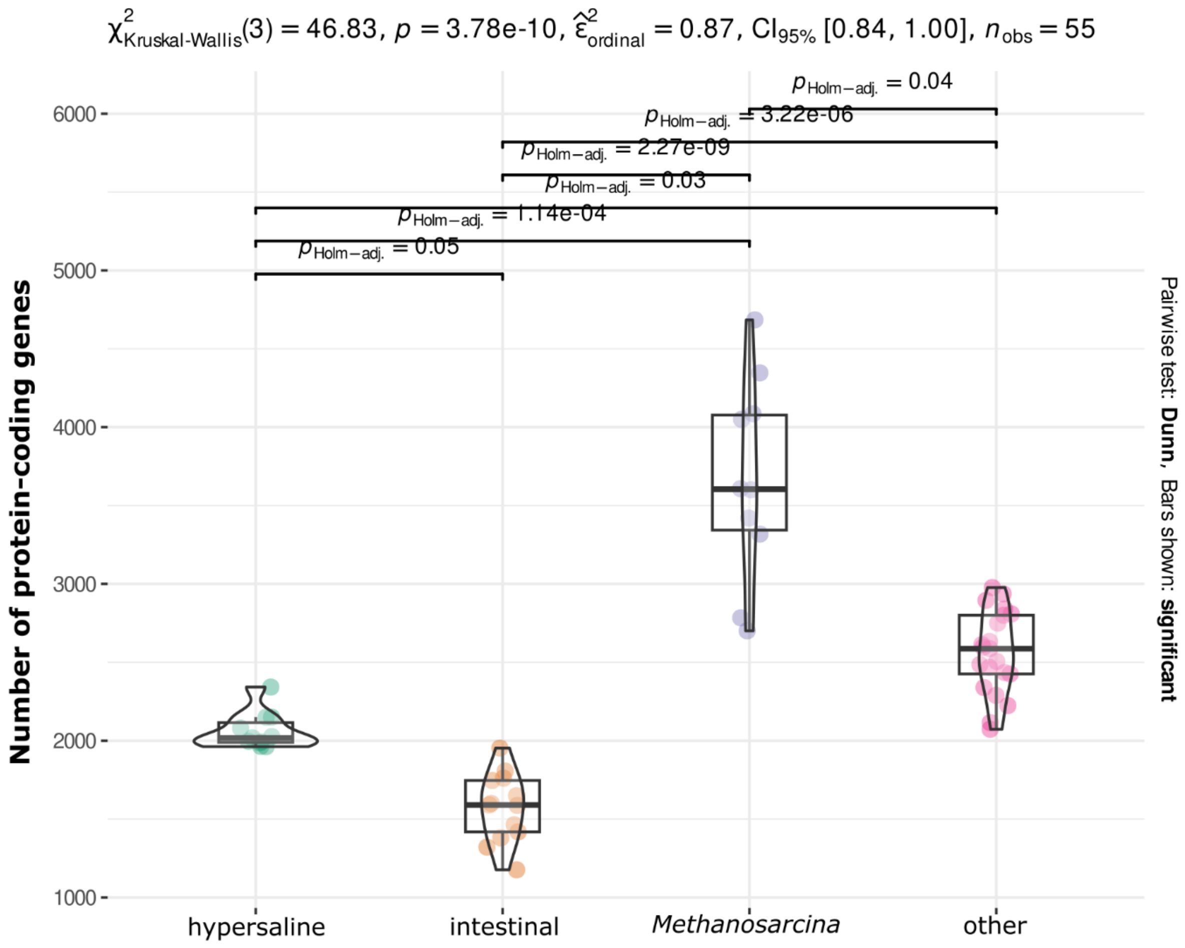

Figure A4. Comparison of number of protein-coding genes between different groups of *Methanosarcinales* (same dataset as in Fig. 6). The numbers of protein-coding genes were not normally distributed (Shapiro–Wilk normality test, $p < 0.05$). Significant differences between the mean values are indicated (Kruskal–Wallis test and post-hoc Dunn tests with Holm p-value adjustment).
